# Around the Hospitals

**Published:** 1895-06-15

**Authors:** 


### AROUND THE HOSPITALS.

[NOTICE TO THE MANAGERS OF INSTITUTIONS.—Special space is now reserved for the insertion of notices of hospital meetings and festivals. The Editor requests that all notices may reach THE HOSPITAL Office, 428, Strand, at least one week before the date of each meeting.]

**The Queen's Hospital, Birmingham.**—This hospital is to be congratulated on its report of the past year. For the first time for some years the subscription list shows an increase. An increase in this direction is most necessary, as the ordinary income is much below the expenditure, which has been increasing for some time. This has not arisen from extravagance, but through the needs of the institution and to secure proper comfort and efficiency throughout. With all due regard to these last two points, economy is maintained in the administration, the cost of maintenance being only 8·7 per cent., though 10 or 12 per cent. is given as a not unfair proportion in "Burdett's Hospital and Charities Annual."

The hospital, which earnestly strives to be abreast of the times, has adopted the uniform system of accounts so strongly advocated by its late general superintendent (Mr. Henry C. Burdett). That the hospital is a growing necessity to the population is proved by the increase of patients applying for admission. During the past year the number of in-patients amounted to 2,190, the largest received since the opening of the hospital. The improvements effected in the operating theatre have proved most satisfactory. The cost was defrayed by the President and Mr. Albright, the latter gentleman also contributing towards the purchase of new instruments. The proposal to establish a dental department has been abandoned owing to lack of space. During the past year the in-patients numbered 2,190, and the out-patients 28,671. The income amounted to £8,401; the expenditure to £8,884; the cost per in-patient, £2 8s. 7d.

